# Environmental Regulation Effect on Green Total Factor Productivity: Mediating Role of Foreign Direct Investment Quantity and Quality

**DOI:** 10.3390/ijerph20043150

**Published:** 2023-02-10

**Authors:** Yusen Luo, Zhengnan Lu, Chao Wu, Claudia Nyarko Mensah

**Affiliations:** 1 School of Management, Jiangsu University, Zhenjiang 212013, China; 2 School of Finance and Economics, Jiangsu University, Zhenjiang 212013, China; 3 Department of Management Studies Education, Akenten Appiah-Menka University of Skills Training and Entrepreneurial Development, Kumasi 00233, Ghana

**Keywords:** green total factor productivity, FDI quantity, FDI quality, China

## Abstract

Green total factor productivity (GTFP) is an excellent index for green development. The objective of this study was to check whether environmental regulation (ER) can affect GTFP through the mediating role of foreign direct investment (FDI) quantity and quality. Using the super-efficiency Epsilon-based measure (EBM) model and a Malmquist–Luenberger (ML) index, China’s GTFP growth was measured during 1998–2018. On this basis, we adopted a Systematic Generalized Method of Moments (SYS-GMM) to analyze the effect of ER on GTFP. The findings show that China’s GTFP declined first and rose again during the sample period. GTFP in the coastland was greater than that in the inland region. ER positively affected China’s GTFP growth. FDI quantity and quality mediated the nexus between ER and GTFP growth in the whole nation. Specifically, this mediation role of FDI quantity and quality was only significant in coastal China. Additionally, financial development can also boost GTFP growth in China. Given the importance of developing a green economy, the government should improve the FDI quality and attract green FDI.

## 1. Introduction

The extensive economic growth mode has caused severe waste pollutant emissions [1], as well as CO_2_ emissions [2]. This has led to catastrophic global warming [3], which will inevitably increase the health risks and shorten life expectancy throughout the world [4]. With the continuous environmental degradation, green development has become a common pursuit for all nations of which China is no exception [5]. The United Nations Development Programme (UNDP) has also pressed China to develop a green economy [6]. Considering the necessity of developing a green economy, the Chinese government has proposed to construct an ecological civilization model since 2007. In addition, the government has formulated several strict environmental policies such as the “Double control zone” as a strategy to practice green development. Through tremendous efforts, China has made remarkable contributions to global sustainability, which is highly praised by the UNDP in 2019 [6].

However, statistics show that the amount of China’s coal consumption and CO_2_ emissions is still on the increase. IEA stated that China is the only major economy that increased fossil fuel demand and reached the highest ever level [7]. As a consequence, China’s CO_2_ emissions in 2021 increased by 6% above 2019 levels. This suggests that China’s green development still has substantial room for improvement [8]. On this premise, evaluating the development level of China’s green economy and its influencing factors has great practical significance, which is also one of the major motives of this study. In line with Wang and Feng [9], we employed GTFP to measure China’s green development, and study its spatial-temporal characteristics.

With the increasing attention paid to global environmental issues, ER is regarded as one of the essential tools for accelerating green economic growth. The nexus among ER and green economic growth has attracted numerous scholars, but the conclusions have failed to reach a consensus. On the one hand, ER exerts an inhibitory effect in promoting green development due to its increased production costs [10]. On the other hand, the promotion effect of ER can compensate for the added environmental expenditure through technology innovation, thereby accelerating the green development [11]. Based on this premises, we propose the first research question as follows: what is the influence of ER on the GTFP?

Additionally, China is diligently practicing the opening strategy; therefore, inflow of foreign direct investment increased dramatically by 20% in 2021 over 2020, reaching a record of 179 billion dollars [12]. However, the effect of FDI on environment is still puzzled. Existing literature has shown evidence that FDI exerts a dual influence, which is either a “Pollution hallo” or a “Pollution haven”, on China’s ecological quality [13]. In most studies, indicators representing the quantity of FDI are used as proxy variables for FDI, such as FDI inflows [14], FDI stock or per capita FDI inflows [15], rather than FDI quality. Meanwhile, strengthening ER significantly affects the FDI inflows [16]. In such an instance, it is as a result of such countries practicing either a “Race to the top” or “Race to the bottom” approach. In the former, local governments in an effort to attract clean industries tighten environmental regulations. In the latter, environmental regulations are laxed for the sake of improving economic development. This results in dirty industries moving into such zones to intensify pollution. The Chinese government has begun to focus on the quality of foreign investment rather than the quantity. In view of this, the following research questions are presented sequentially: does ER expand China’s GTFP through the intermediary role of FDI and is it more effective to focus on the quality of FDI rather than the quantity?

To seek answers to the above questions, it is of great practical significance to check whether ER can affect GTFP through the mediating role of FDI quantity and quality, which provides empirical evidence for other developing countries to formulate ER and opening policies. This study contributes to erstwhile literature in the following manner. Firstly, this study employed the metafrontier super-efficiency EBM model with undesirable output (Undesirable-M-Super-EBM) and ML index to construct a more accurate assessment of China’s GTFP. Compared with the traditional radial and non-radial DEA models, the Undesirable-M-Super-EBM model contains slack variables and allows the input variables to change proportionally, which improves the accuracy of productivity measurement. Secondly, this paper probed into the influencing channels through which ER affects GTFP. The existing research mainly focuses on the mediation role of FDI quantity when analyzing the indirect effect of environmental regulation. However, few scholars have also looked at the mediating role of FDI quality. Thus, this paper measured the quality of FDI from the following four perspectives: FDI’s performance, FDI’s unit scale, FDI’s export capacity and FDI’s technological spillover, and checked the mediating role of FDI quality through environmental regulation on GTFP. Last, taking into consideration the huge regional heterogeneities, this paper performed heterogeneity analysis and investigated the influences of ER on GTFP in coastal and inland areas.

The paper is structured as follows. The related literature is reviewed in Section 2. Methodology, variable selection and data sources are presented in Section 3. Section 4 provides the estimated GTFP and the regression results. Section 5 presents the conclusion and policy suggestions.

## 2. Theoretical Background and Hypothesis Development

### 2.1. Environmental Regulation and GTFP

There have been three mainstream theories on the ER–GTFP nexus in the extant literature. The first theory is the “Compliance cost hypothesis”, postulating that the strengthening of ER intensity will constrain improvement in GTFP [10]. Increased environmental expenditure caused by ER will raise the enterprises’ production cost and bring down R&D activities. Consequently, reducing the profit margins and technological innovation capacity [17]. Ni et al., also argued that the introduction of a carbon emission trading system increased the firms’ cost debt and distress risk [18].

On the other hand, the “Porter hypothesis” proposed by Porter held the opposite view [19]. Porter opined that intensifying ER can stimulate innovation and promote TFP growth [19], and his opinion was confirmed by several studies. Zhuo et al., also validated the existence of “Porter hypothesis” [11]. The “Porter hypothesis” is further extended to weak and narrow versions [20]. The “weak” Porter hypothesis has been verified in China’s Cleaner Production Audit program. It found that environmental regulation has a positive effect on green innovation [21], which can help improve enterprise performance [22]. The narrow version stated that flexible ER can stimulate a firm to innovate and promote productivity [20].

The debate on the “Compliance cost hypothesis” and “Porter hypothesis” also yields the third school of thought, which claims that a nonlinear relation is exists between ER and GTFP [23]. The degree of domination of ER on GTFP is dependent on the resultant force of “Porter’s hypothesis effect” and “Compliance cost effect”. Based on the above literature, we developed the first hypothesis as follows.

**Hypothesis** **1.** *ER exerts a direct promotion effect on GTFP growth*.

### 2.2. FDI and GTFP

The literature on the nexus between FDI and GTFP growth can trace back to two famous hypotheses, namely the “Pollution halo” and “Pollution haven” hypothesis [4,24]. The Pollution Halo hypothesis claims that advanced technology and management skills are introduced to host countries with the inflow of FDI [25]. These can improve the environmental quality such host countries [4] and this has been confirmed in the literature. Specifically in China, Fang et al., found that FDI played a significant “Pollution halo” effect [26].

The Pollution Haven hypothesis postulates that high polluting industries are inclined to transfer to regions with lax environmental standards, which might worsen the host countries’ environmental quality and inhibit GTFP growth [24]. In the context of China, Wang et al., validated the “Pollution haven” effect of FDI by adopting spatial econometric model [27].

On this premises, the second hypothesis was developed as follows.

**Hypothesis** **2.** *FDI can promote GTFP growth*.

### 2.3. Environmental Regulation, FDI and GTFP

**Figure 1:**
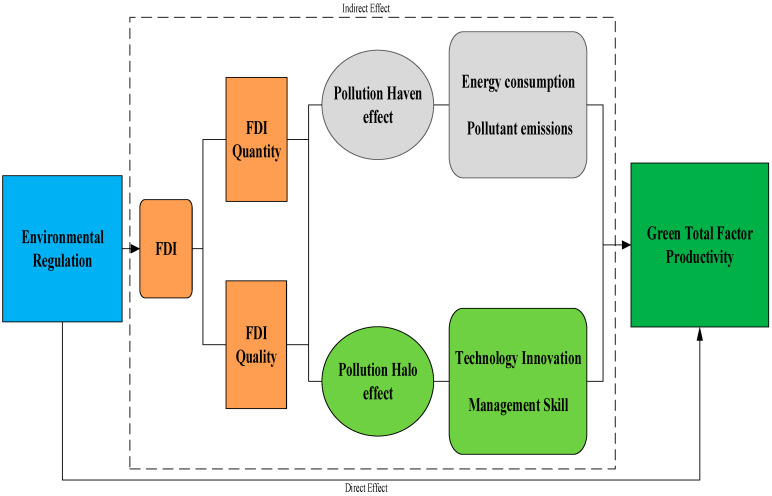
The theoretical framework of ER on China’s GTFP growth.

The theoretical frame of ER on GTFP is displayed in Figure 1. It depicts that ER can not only directly affect GTFP growth, but can also determine GTFP growth through diverse channels, of which FDI is one of the most important. As seen in Figure 1, strengthening regulatory tools comes with the tendency to raise the FDI access threshold, which might result in a decrease in FDI quantity [28], as well as foreign technology spillover. The international enterprises are also inclined to transfer their industries to those countries with lower environmental standards. To promote economic growth, some developing countries compete to attract grey FDI and promote economic growth through ease-going on ecological standards, known as “Race to the bottom hypothesis” [29]. In such scenarios, FDI might increase the energy consumption and exert a pollution haven effect on pollutants, which will eventually restrain GTFP growth.

Contrary to this, the local government might also compete to intensify ER to pull high-quality FDI, which will expand GTFP growth [28]. It is known as the “Race to the top hypothesis”. Moreover, as an important policy instrument, the strengthening of ER also indicates the improvement in the quality of the national system, while FDI tends to flow into countries with better institutional quality [30]. Some studies, e.g., Yu and Li [31], noted that the stricter ER can draw clean FDI from developed countries. Moreover, with the increase in FDI quality, its pollution halo effect will accelerate the technological innovation and improve the management skills, which significantly contribute to the GTFP growth.

Based on the, the third hypothesis is proposed.

**Hypothesis** **3a.** *FDI quantity is a mediating path of ER and GTFP growth*.

**Hypothesis** **3b.** *FDI quality is a mediating path of ER and GTFP growth*.

## 3. Method, Variables and Data

In this section, we firstly introduce the undesirable super-EBM model under metafrontier and ML index, which is widely used to measure GTFP growth [32]. Then, the variable selection and data resources are also presented. Moreover, the econometric model and mediation model is constructed to analyze the mediating role of FDI in the influencing channel of ER on GTFP.

### 3.1. Measurement of GTFP 

#### 3.1.1. Environmental Technology under Metafrontier

On a production line, companies or organizations place factors, such as labor, capital and energy into production for increasing desirable output, e.g., GDP. However, according to null jointness theory, the undesirable outputs, e.g., carbon dioxide emissions (CO_2_) always coexist with the good output due to the consumption of energy [33]. So, the production possibility set *P*(*x*) was constructed as follows:

(1) $$P\left(x\right)=\left\{\left(y,b\right):x\to \left(y,b\right)\right\},x\in R_{M},y\in R_{R},b\in R_{V}$$

In Equation (1), *x*, *y* and *b* are input, desirable and undesirable output matrixes. In line with Luo et al. [32], Equation (1) can be further modeled to be Equation (2) as follows.

(2) $$P^{h}=\left\{\left(x^{h},y^{h},b^{h}\right):\sum_{n=1}^{N_{h}}\xi_{n}^{h}x_{n}^{h}\leq x^{h},\sum_{n=1}^{N_{h}}\xi_{n}^{h}y_{n}^{h}\geq y^{h},\sum_{n=1}^{N_{h}}\xi_{n}^{h}b_{n}^{h}\leq b^{h}\right\}$$

The production possibility set defined in Equation (1) assumed that all DMUs (decision making units) have the same production technology. However, in the reality, due to the heterogeneity of the factor endowments and economic growth among different provinces, the production technology might be different. Hence, we follow the study of Luo et al. [34], and define the environmental technology under metafrontier. We divide China’s provinces into *H* technology groups. The production set under metafrontier *P^meta^* is described in Equation (3).

(3) $$P^{meta}=\left\{\left(x,y,b\right):\sum_{h=1}^{H}\sum_{n=1}^{N_{h}}\lambda_{n}^{h}x_{n}^{h}\leq x^{h},\sum_{h}^{H}\sum_{n=1}^{N_{h}}\lambda_{n}^{h}y_{n}^{h}\geq y^{h},\sum_{h}^{H}\sum_{n=1}^{N_{h}}\lambda_{n}^{h}b_{n}^{h}\leq b^{h}\right\}$$

where $\lambda_{n}^{h}\geq 0,n=1,2,\cdots ,N_{h}$, $P^{meta}=P^{1}\cup P^{2}\cup \cdots \cup P^{H}$.

#### 3.1.2. Undesirable-Super-EBM Model

GTFP is extensively measured by the DEA model. The traditional radial models, e.g., CCR, might neglect the non-radial slacks, which will overvalue the efficiency score. Meanwhile, the non-radial models, e.g., SBM model, may fail to change proportionality and lead to a biased estimation [35]. To solve the shortcomings of the above DEA models, Tone and Tsutsui introduced the EBM model [35], which is widely used by many researchers [8,36]. Hence, this study takes on EBM-DEA to measure Chinese GTFP from 1998 to 2018.

Although the EBM model has made significant progress compared to the CCR and SBM models, there are some problems that need to be solved in the measurement of GTFP. The original EBM model failed to take the undesirable output into consideration. It cannot discriminate the efficiency scores that are equivalent to 1. Hence, following the study of Luo et al. [8], Undesirable-Super-EBM under group frontier and metafrontier are defined in Equations (4) and (5).

(4) $$\begin{array}{l} \min\rho_{{}_{\mathrm{ko}}}^{\mathrm{group}}=\frac{\theta -\varepsilon_{x}\frac{1}{\sum_{m=1}^{M}w_{mo}^{x}}\sum_{m=1}^{M}\frac{w_{mo}^{x}s_{mo}^{x}}{x_{mo}}}{\varphi +\left(\varepsilon_{y}\frac{1}{\sum_{r=1}^{R}w_{ro}^{y}}\sum_{r=1}^{R}\frac{w_{ro}^{y}s_{ro}^{y}}{y_{ro}}+\varepsilon_{b}\frac{1}{\sum_{v=1}^{V}w_{vo}^{b}}\sum_{v=1}^{V}\frac{w_{vo}^{b}s_{vo}^{b}}{b_{vo}}\right)}\\ s.t.\;\sum_{n=1,n\neq o}^{N}\lambda_{n}x_{mn}=\theta x_{mo}-s_{mo}^{x};\\ \sum_{n=1,n\neq o}^{N}\lambda_{n}y_{rn}=\varphi y_{ro}+s_{ro}^{y}\\ \sum_{n=1,n\neq o}^{N}\lambda_{n}b_{vn}=\varphi b_{vo}-s_{vo}^{b};\\ 0<s_{mo}^{x}\leq 1;0<s_{ro}^{y}\leq 1;0<s_{vo}^{b}\leq 1;\sum \lambda =1;\\ 0<\theta \leq 1;\varphi \geq 1;0<\varepsilon_{x}\leq 1;0<\varepsilon_{y}\leq 1;0<\varepsilon_{b}\leq 1;\\ m=1,2,\cdots ,M;r=1,2,\cdots ,R;v=1,2,\cdots ,V. \end{array}$$

(5) $$\begin{array}{l} \min\rho_{ko}^{meta}=\frac{\theta -\varepsilon_{x}\frac{1}{\sum_{m=1}^{M}w_{mko}^{x}}\sum_{m=1}^{M}\frac{w_{mko}^{x}s_{mko}^{x}}{x_{mko}}}{\varphi +\left(\varepsilon_{y}\frac{1}{\sum_{r=1}^{R}w_{rko}^{y}}\sum_{r=1}^{R}\frac{w_{rko}^{y}s_{rko}^{y}}{y_{rko}}+\varepsilon_{b}\frac{1}{\sum_{v=1}^{V}w_{vko}^{b}}\sum_{v=1}^{V}\frac{w_{vko}^{b}s_{vko}^{b}}{b_{vko}}\right)}\\ s.t.\;\sum_{h=1}^{H}\sum_{n=1,n\neq oifh=k}^{N_{h}}\lambda_{n}^{h}x_{mhn}=\theta x_{mko}-s_{mko}^{x};\\ \sum_{h=1}^{H}\sum_{n=1,n\neq oifh=k}^{N_{h}}\lambda_{n}^{h}y_{rhn}=\varphi y_{rko}+s_{rko}^{y};\\ \sum_{h=1}^{H}\sum_{n=1,n\neq oifh=k}^{N_{h}}\lambda_{n}^{h}b_{vhn}=\varphi b_{vko}-s_{vko}^{b};\\ 0<s_{mko}^{x}\leq 1;0<s_{rko}^{y}\leq 1;0<s_{vko}^{b}\leq 1;\sum \lambda =1;\\ 0<\theta \leq 1;\varphi \geq 1;0<\varepsilon_{x}\leq 1;0<\varepsilon_{y}\leq 1;0<\varepsilon_{b}\leq 1;\\ m=1,2,\cdots ,M;r=1,2,\cdots ,R;v=1,2,\cdots ,V. \end{array}$$

whereas $\rho_{ko}^{group}$ and $\rho_{ko}^{meta}$ are the efficiency values measured by Undesirable-Super-EBM model under group-frontier and metafrontier. *x_mko_*, *y_rko_*, and *b_vko_* are respectively inputs, desirable, and undesirable outputs. $s_{mko}^{x}$, $s_{rko}^{y}$ and $s_{vko}^{b}$ are the slacks of the *x_mko_*, *y_rko_*, and *b_vko_*; $w_{mko}^{x}$, $w_{rko}^{y}$ and $w_{vko}^{b}$ are the corresponding weights of input–output variables. *ε_x,y,b_* is the weight of non-radial part in the EBM model. ∑λ=1 indicates that the estimation is under variable return to scale (VRS). In addition, the technology gap ratio (TGR), under the interval of (0, 1], is calculated based on Equation (6).

(6) $$TGR=\frac{\rho_{ko}^{meta}}{\rho_{ko}^{group}}$$

#### 3.1.3. ML Index

In line with Luo et al. [8], the ML index under group frontier and metafrontier are constructed to measure GTFP growth (see Equations (7) and (8)).

(7) $$\begin{array}{ll} ML_{t}^{t+1,group} & =\sqrt{\frac{D^{t,group}\left(x^{t+1},y^{t+1},b^{t+1}\right)}{D^{t,group}\left(x^{t},y^{t},b^{t}\right)}\times \frac{D^{t+1,group}\left(x^{t+1},y^{t+1},b^{t+1}\right)}{D^{t+1,group}\left(x^{t},y^{t},b^{t}\right)}}\\ & =\frac{D^{t+1,group}\left(x^{t+1},y^{t+1},b^{t+1}\right)}{D^{t,group}\left(x^{t},y^{t},b^{t}\right)}\times \sqrt{\frac{D^{t,group}\left(x^{t},y^{t},b^{t}\right)}{D^{t+1,group}\left(x^{t},y^{t},b^{t}\right)}\times \frac{D^{t,group}\left(x^{t+1},y^{t+1},b^{t+1}\right)}{D^{t+1,group}\left(x^{t+1},y^{t+1},b^{t+1}\right)}}\\ & =EC_{t}^{t+1,group}\times TC_{t}^{t+1,group} \end{array}$$

(8) $$\begin{array}{ll} ML_{t}^{t+1,meta} & =\sqrt{\frac{D^{t,meta}\left(x^{t+1},y^{t+1},b^{t+1}\right)}{D^{t,meta}\left(x^{t},y^{t},b^{t}\right)}\times \frac{D^{t+1,meta}\left(x^{t+1},y^{t+1},b^{t+1}\right)}{D^{t+1,meta}\left(x^{t},y^{t},b^{t}\right)}}\\ & =\frac{D^{t+1,meta}\left(x^{t+1},y^{t+1},b^{t+1}\right)}{D^{t,meta}\left(x^{t},y^{t},b^{t}\right)}\times \sqrt{\frac{D^{t,meta}\left(x^{t},y^{t},b^{t}\right)}{D^{t+1,meta}\left(x^{t},y^{t},b^{t}\right)}\times \frac{D^{t,meta}\left(x^{t+1},y^{t+1},b^{t+1}\right)}{D^{t+1,meta}\left(x^{t+1},y^{t+1},b^{t+1}\right)}}\\ & =\frac{D^{t+1,group}\left(x^{t+1},y^{t+1},b^{t+1}\right)}{D^{t,group}\left(x^{t},y^{t},b^{t}\right)}\times \frac{TGR^{t+1}\left(x^{t+1},y^{t+1},b^{t+1}\right)}{TGR^{t}\left(x^{t},y^{t},b^{t}\right)}\times \sqrt{\frac{D^{t,meta}\left(x^{t},y^{t},b^{t}\right)}{D^{t+1,meta}\left(x^{t},y^{t},b^{t}\right)}\times \frac{D^{t,meta}\left(x^{t+1},y^{t+1},b^{t+1}\right)}{D^{t+1,meta}\left(x^{t+1},y^{t+1},b^{t+1}\right)}}\\ & =EC_{t}^{t+1,group}\times \frac{TGR^{t+1}\left(x^{t+1},y^{t+1},b^{t+1}\right)}{TGR^{t}\left(x^{t},y^{t},b^{t}\right)}\\ & \times \sqrt{\frac{D^{t,group}\left(x^{t},y^{t},b^{t}\right)}{D^{t+1,group}\left(x^{t},y^{t},b^{t}\right)}\times \frac{D^{t,group}\left(x^{t+1},y^{t+1},b^{t+1}\right)}{D^{t+1,group}\left(x^{t+1},y^{t+1},b^{t+1}\right)}\times \frac{TGR^{t}\left(x^{t+1},y^{t+1},b^{t+1}\right)}{TGR^{t+1}\left(x^{t},y^{t},b^{t}\right)}\times \frac{TGR^{t}\left(x^{t},y^{t},b^{t}\right)}{TGR^{t+1}\left(x^{t+1},y^{t+1},b^{t+1}\right)}}\\ & =EC_{t}^{t+1,group}\times TC_{t}^{t+1,group}\times \sqrt{\frac{TGR^{t+1}\left(x^{t+1},y^{t+1},b^{t+1}\right)}{TGR^{t+1}\left(x^{t},y^{t},b^{t}\right)}\times \frac{TGR^{t}\left(x^{t+1},y^{t+1},b^{t+1}\right)}{TGR^{t}\left(x^{t},y^{t},b^{t}\right)}}=ML_{t}^{t+1,group}\times TGRC \end{array}$$

where *D^t^*(*x^t^*, *y^t^*, *b^t^*) denotes the distance function of period *t*. *ML_t_^t^*^+1^ is the GTFP change from period *t* to *t* + 1. When *ML_t_^t^*^+1^ > 1, GTFP increases, and vice versa. Additionally, *ML_t_^t^*^+1^ can be disintegrated to *TC_t_^t^*^+1^ and *EC_t_^t^*^+1^. According to Liu and Xin [37], the TC (technology progress change) index refers to the improvement in production technologies, while EC (technological efficiency change) index refers to the development of the production factor allocation method and management system. When the TC (EC) index is greater than 1, it indicates that the production technology (production factor allocation methods) is improved and vice versa. TGRC measures the ratio of ML under the meta and group-frontier. When the TGRC index is approaching 1, it indicates the technology gap between the meta and group-frontier is narrowing.

#### 3.1.4. Input–Output Variables

The green economic development can be regarded as an input–output production process. The selection of the input and output variables is presented in Table 1. In line with the study of Luo et al. [34], we take the capital stock, employment, and energy consumption as input variables. Among these, capital stock refers to the fixed asset investment, which is measured by the perpetual inventory method [38]. Moreover, energy is also the key production factor of economic growth. We follow Zhao et al. [39] and use the total consumption of terminal fossil energy to measure it. Regarding the output variables, it can be divided into two groups: desired output and undesired output. The null jointness theory holds that undesired output always coexists with desired output [33]. Consistent with Luo et al. [34], GDP is selected as the desirable output, while CO_2_ emissions is used as the bad output.

### 3.2. Variable, Econometric Model and Estimation Technique

#### 3.2.1. Variable Selection and Data Sources

(1) Dependent variable. The ML index measured by the super efficiency EBM-ML model under metafrontier is used to proxy the GTFP growth.
(2) Key variable. Considering a single indicator is hard to describe the real environmental regulation intensity [41], following Luo et al. [32], we evaluated ER intensity from the perspective of heterogeneous regulatory tools. Environmental regulation can be categorized into three types, namely command-and-control (CR), market-incentive (MR) and voluntary (VR). The indicators for evaluating ER intensity are consistent with the evaluation index system in Luo et al. [32].
(3) Mediation variables. In line with Wang and Luo [42], FDI quantity (*FDIM*_it_) is calculated by the ratio of FDI to GDP. Evaluating FDI quality is more complex. Researchers are unanimous on the proxied variable of FDI quality. In the view of Wang and Luo [42], FDI quality mainly refers to the management strength and technological level of foreign capital, which can proxy by FDI performance: Pan et al., pointed out FDI quality is an indicator to reflect the technology spillover to the host countries [43]. Yu and Li used the unit scale and unit benefit to evaluate it [31]. Hu and Xu used FDI export capacity as one of the key indicators to evaluate FDI quality [44]. Based on the above study, this paper uses the entropy method and measures FDI quality from the following four dimensions: FDI performance = (*FDI_it_/FDI_t_*)/(*GDP_it_/GDP_t_*); FDI unit scale = actual use of FDI/the number of foreign-funded enterprises; FDI export capacity: FDI industry exports/total regional exports; FDI technological spillover: $\frac{FDI_{it}}{\sum_{i=1}^{30}FDI_{it}}\sum_{j=1}^{22}\frac{FDI_{jt}}{\sum_{j=1}^{22}FDI_{jt}}S_{jt}^{D}$ whereas *S_jt_^D^* denotes the R&D stock of *j* country at period *t*; *FDI_it_* represents the FDI stock of *i* province at period *t* in China; *FDI_jt_* is the FDI stock from *j* country to China. This paper chooses 22 countries in OECD, including America, England, Japan, German, France, Sweden, Canada, Austria, Turkey, Czech Republic, Belgium, Denmark, Greece, Finland, Ireland, Norway, Portugal, Netherlands, Spain, Hungary, South Korea, and Poland as the main sources of FDI technology spillover. The reason is that the technological innovation capacity of these countries is in the forefront of the world. Their R&D capital accounts for nearly 70% of the world’s total R&D capital.
(4) Control variables. *Fine_it_* is calculated by the ratio of loans to deposits [45]. The improvement of *Fine_it_* might stimulate the usage of high energy consumption products, e.g., air conditioners, and increase environmental pollution [46]. On the contrary, financial development efficiency when increased can alleviate funding restriction to stimulate technological innovation, which can reduce R&D risks, and promote GTFP growth [47]. *Gov_it_* is proxied by the ratio of general financial expenditure to GDP [8]. The GDP-driven-styled government intervention might deteriorate environmental quality and inhibit GTFP growth, while the environment-driven-styled intervention can improve GTFP [48]. *Infra_it_* is estimated by the proportion of post and telecommunications business to GDP [49]. Infrastructure construction may increase the use of cement, steel and other materials, which will increase energy consumption and pollutants. On the other side, the improvement of infrastructure may increase energy efficiency and promote GTFP growth [50].

This study adopts the dataset of 30 Chinese provinces during 1998–2018. Due to the data availability, Tibet, Hongkong, Macaw and Taiwan are excluded. The data are arranged from “China Statistical Yearbook”, “China Environment Yearbook”, “OECD Statistics” and “China Energy Statistical Yearbooks”. The descriptive statistics of the selected variables are exhibited in Table 2.

#### 3.2.2. Dynamic Panel Model

The purpose of this study is to study the influence of ER on China’s GTFP growth. The baseline econometric model was constructed as shown below.

(9) $$GTFP_{it}=\alpha_{0}+\alpha_{1}ER_{it}+\beta X_{it}+\mu_{i}+\varepsilon_{it}$$

Considering the current GTFP growth might be impacted by the previous TFP growth [29], we lengthened Equation (9) by introducing the first-order lag variable of GTFP to analyze the cumulative effect of GTFP growth. Thus, the dynamic panel model is expressed in Equation (7).

(10) $$GTFP_{it}=\alpha_{0}+\alpha_{1}GTFP_{i(t-1)}+\alpha_{2}ER_{it}+\beta X_{it}+\mu_{i}+\varepsilon_{it}$$

where *GTFP_it_* denotes the green TFP of *i* province at period *t*. *ER_it_* denotes environmental regulation of *i* province at period *t*. *X_it_* is the control variable matrix, which contains financial development efficiency (*Fine_it_*), governmental intervention (*Gov_it_*) and infrastructure level (*Infra_it_*). *μ_i_*, and *ε_it_* represent the individual effect and random error, respectively.

#### 3.2.3. Mediation Model

To investigate the mediating role of FDI quantity and quality through the influencing channel of ER on GTFP growth, this paper follows the study of Baron and Kenny [51], and constructs the mediation models as follows.

(11) $$FDIM_{it}=\gamma_{0}+\gamma_{1}FDIM_{i(t-1)}+\gamma_{2}ER_{it}+\beta X_{it}+\mu_{i}+\varepsilon_{it}$$

(12) $$GTFP_{it}=\tau_{0}+\tau_{1}GTFP_{i(t-1)}+\tau_{2}ER_{it}+\tau_{3}FDIM_{it}+\beta X_{it}+\mu_{i}+\varepsilon_{it}$$

(13) $$FDIQ_{it}=\gamma_{0}+\gamma_{1}FDIQ_{i(t-1)}+\gamma_{2}ER_{it}+\beta X_{it}+\mu_{i}+\varepsilon_{it}$$

(14) $$GTFP_{it}=\tau_{0}+\tau_{1}GTFP_{i(t-1)}+\tau_{2}ER_{it}+\tau_{3}FDIQ_{it}+\beta X_{it}+\mu_{i}+\varepsilon_{it}$$

where *FDIM*_it_ and *FDIQ*_it_ denote FDI quantity and FDI quality.

#### 3.2.4. SYS-GMM Estimation

Considering the lagged term of dependent variable (*GTFP_it_*) might lead to endogenous problems, we adopt the SYS-GMM technique to overcome this problem. The GMM method was firstly proposed by Arellano and Bond [52]. It introduced the level values of dependent variables as instrumental variables to overcome the endogenous problem. However, Blundell and Bond [53] point out that the first-order difference GMM estimation method is easy to be affected by weak instrumental variables and lead to biased estimation results. To avoid the influence of weak instrumental variables, Arellano and Bover [54], Blundell and Bond [53] pointed out that not only the level variable of the dependent variable but also the lagged value of the difference variable can be used as the additional instrumental variable in the GMM estimation, that is, the system GMM method. It combined the level regression equation and the difference regression equation to estimate. Thus, the SYS-GMM technique is employed in this study.

## 4. Empirical Results and Discussion

### 4.1. Results of China’s GTFP Growth

**Figure 2:**
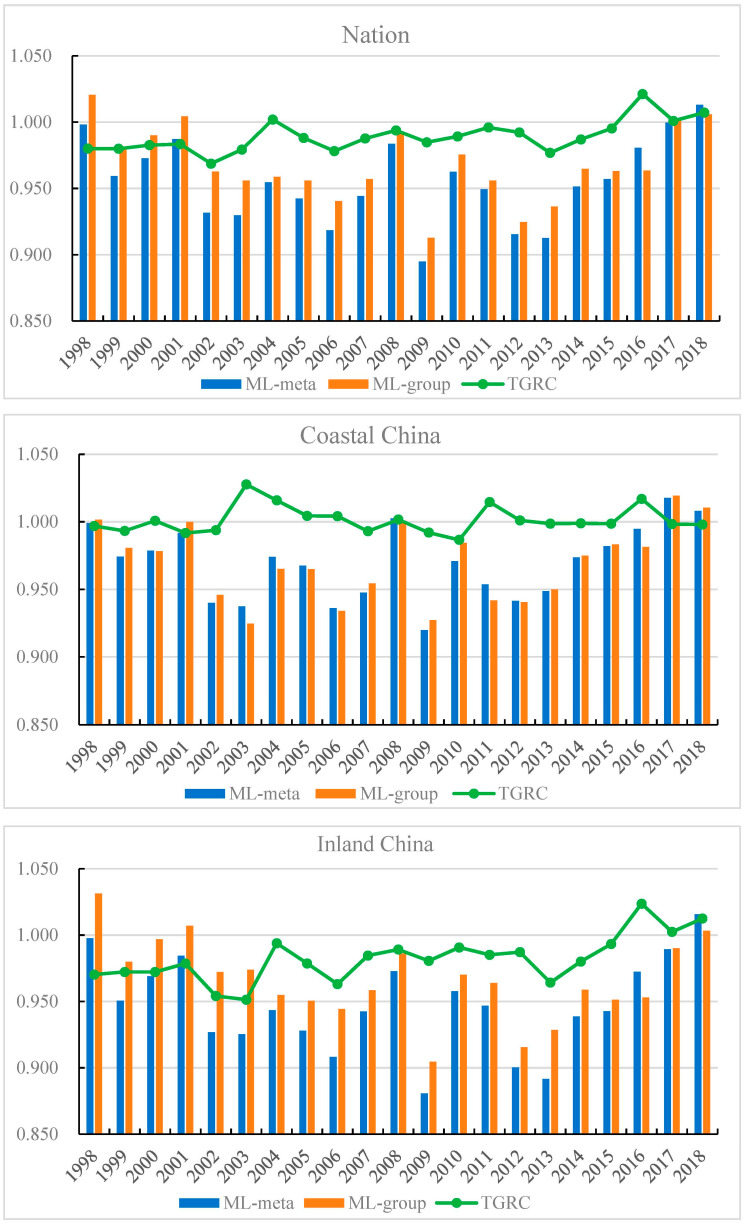
Trend of ML-meta, ML-group and TGRC index in different regions.

Based on the Undesirable-Super-EBM model, the ML index of Chinese 30 provinces during 1998–2018 was calculated. Figure 2 displays the evolutionary tendency of the ML index under metafrontier and group frontier. It can be seen that both the ML-meta and ML-group indexes in terms of whole nation fluctuated between 1998 and 2013, then increased dramatically from 2013 to 2018. The ML-meta and ML-group indexes were lowest in the year of 2009. The average ML index under metafrontier and group frontier during 1998–2018 was 0.955 and 0.968, illustrating that the green development in China has substantial room for improvement. Moreover, the TGRC index exhibited an upward tendency, indicating the technological gap between metafrontier and group frontier has narrowed during 1998–2018. 

This study further compared the ML-meta index in coastal area and inland area (see Figure 2). It showed that coastal area had the largest ML-meta index (0.969) during the sample period, followed by inland area (0.947). Additionally, the TGRC index in coastland was greater than that in inland, implying that the actual production technology in coastal provinces was closer to the potential optimal technology level than inland provinces. From 2013 to 2018, the ML-meta index in inland area increased at the rate of 2.64%, which is faster than that in the coastal area (1.22%). It indicates that there a convergence characteristic of GTFP growth in China might exist. 

**Figure 3:**
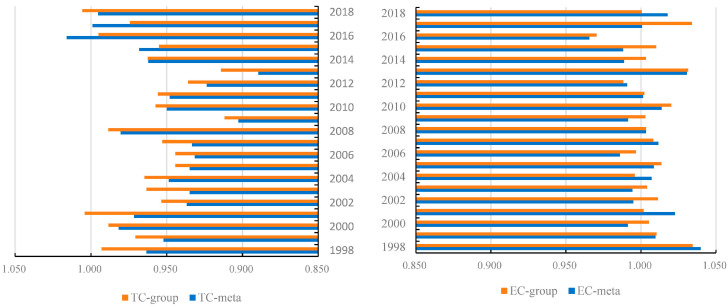
Trend of EC and TC indexes under metafrontier and group frontier in China.

In Figure 3, the trends of ML, EC and TC indexes under metafrontier and group frontier are compared. It depicts that the average EC group was larger than EC meta. Additionally, in most periods, the EC index was obviously larger than the TC index under the corresponding production technology set. The average EC index with respect to metafrontier was 1.003 and remained stable from 1998 to 2018. It indicates that the China had optimal production factor allocation. Regarding the TC index, the average value of TC meta and TC group from 1998 to 2018 was 0.953 and 0.964, respectively. TC meta exceeded 1 only in the year of 2016, while TC group was larger than 1 in 2001 and 2018, implying that China achieved green technical progress in these years.

This paper also calculated the average ML-meta index of each province (See Table 3). It can be found that Beijing had the largest ML-meta index (1.009), followed by Shanghai (0.998) and Guangdong (0.992). By contrast, the ML index measured by metafrontier in Gansu (0.856) and Ningxia (0.925) was smallest. The EC index in coastal provinces was similar to that in inland provinces, while the coastal area had a higher TC index. Hence, it is imperative for inland provinces to develop green technologies.

### 4.2. Multiple Collinearity Test

Considering the fact that a multicollinearity problem might cause the regression results to be biased, we followed the study of Wang and Luo [42], and adopted the variance expansion factor (VIF) to affirm whether the selected variables are highly correlated. Table 4 reports multiple collinearity results. The largest VIF value is 1.81, suggesting that no serious multicollinearity issue existed in the model.

### 4.3. Stationary Test

To avoid the biased estimation caused by false regression, we also employed Im–Pesaran–Shin, Breitung and Augmented Dickey–Fuller tests to assess the stationarity of the time series. The null hypotheses of the three unit root tests in all the panels contain a unit root. As seen in Table 5, the *p* values of some variables, e.g., *Fine_it_*, *Gov_it_*, are larger than 0.1, which indicates that they cannot reject the null hypotheses at level. In other words, the stationarity of their sequence cannot be confirmed. However, the results suggest that the statistics of all variables are significant at the 1% significance level after taking the first difference. Hence, the null hypothesis that the data are nonstationary is rejected. 

In line with Lin and Chen [55], this paper further used cointegration tests (Kao, Pedroni and Westerlund) to analyze whether the variables had equilibrium nexus. In Table 6, it is observed that all the *p* values are below 0.1, indicating that a long-term cointegration relation exists among GTFP growth and its determinants. 

### 4.4. SYS-GMM Results

#### 4.4.1. Direct Effect of ER on China’s GTFP

Table 7 reports the direct influence of ER on China’s GTFP. Clearly, the AR (1) tests in each table are significant at the 5% level, which suggests that the first-order error term in all regressions are autocorrelated. The AR (2) test and Hansen test points that there is no second-order serial correlated and IVs are not overidentified. In other words, the estimated results based on SYS-GMM are effective. Additionally, the lagged GTFP positively impacted the current GTFP at the 1% confidence level with the coefficient value of 0.259. It implies that the improvement in China’s GTFP has a cumulative effect [29].

ER exerts a significant and positive influence on GTFP growth in terms of whole nation. The stricter ER might force firms to strengthen technological innovation, which can offset the compliance costs and promote GTFP growth [19,32]. In coastal region, the coefficient of *ER_it_* is 0.038, and significant at 10% level. However, in Inland China, the effect of ER is negative (−0.011). Moreover, *Fine_it_* positively boosted China’s GTFP growth at the 10% level, indicating that the development of finance can expand innovation and productivity.

For the validity of the estimation results above, robust analysis was carried out by changing the estimation method. Considering the data distribution of the GTFP growth is truncated, the estimation results based on SYS-GMM might lead to bias. Hence, we employed the Tobit regression model and Truncated regression model to investigate the nexus among ER and GTFP growth in China. It is observed that the influence of *ER_it_* on GTFP is positive at the 5% level, confirming the results based on SYS-GMM. Moreover, we also used the first and the second lagged term of ER to replace *ER_it_*, and re-estimate the impact of ER on GTFP growth. The results in Table 8 confirmed the promotion effect of ER again.

#### 4.4.2. Mediation Effect of FDI Quantity

Table 8 reports the mediating role of FDI quantity on GTFP growth through ER. It was found that Arellano–Bond test and Hansen test suggest the estimation results based on SYS-GMM are reasonable. From the results on the entire nation, both FDI quantity and GTFP growth exert cumulative effects at the 1% significance level. Environmental regulation intensity positively affects FDI quantity at the 1% level, which agrees with the results of Yu and Li [31]. Intensifying environmental regulation implies the improvement of institutional environment quality, which may attract more FDI inflow [30]. Meanwhile, FDI quantity also exerts a significant “Pollution halo” effect on China’s GTFP. FDI inflow brings the advanced technologies as well as the environmental management skills into China [56], which can decrease the pollutant emissions and promote GTFP growth. Hence, FDI quantity mediates the relationship between GTFP growth and ER.

In the coastal model, the coefficient of *ER_it_* on *FDIM_it_* is significantly positive, while the influence of *FDIM_it_* on *GTFP_it_* is positive at the 10% significance level, indicating that FDI quantity can explain the relationship between ER and FDIM in coastal area. In the inland area, the influence of ER on FDI quantity is positive but fails to pass the statistical confidence test, implying that the mediating role of FDI quantity is not significant. 

Regarding the control variables, *Fine_i_*_t_ exerts a positive role on FDI quantity with the coefficient of 0.232, which agrees with the study of Chen et al. [47]. The improvement in financial efficiency can expand external financing channels and support economic activities, which will attract FDI inflows. In the coastal area, the coefficient of *Gov_i_*_t_ on GTFP is 0.383 and statistically significant. It suggests that the governmental performance evaluation system is environment-driven in coastal provinces [32]. In the inland model, governmental intervention positively affects the FDI quantity at the 5% level.

For the robustness of the conclusions drawn above, Sobel test, Aroian test and Goodman test were employed to examine the mediation effect of FDI quantity in the impacting channel of ER on green TFP growth. The results of the Sobel test, Aroian test and Goodman test are 1.817, 1.759 and 1.880, confirming the validity that FDI quantity has a significant mediation effect.

#### 4.4.3. Indirect Effect of ER on China’s GTFP: Mediation Effect of FDI Quality

Similarly, we also tested the mediation role of FDI quality based on the model proposed by Baron and Kenny [51]. The results are reported in Table 9. This study found that stricter environmental regulation can improve FDI quality at the national level and also in coastal areas. Attracting more FDI quality can also exert technology spillover and demonstration effects on the local enterprises, consequently promoting green productivity growth. To sum up, in terms of whole nation and coastal area, FDI quality also mediates the relationship between ER and GTFP growth. In inland provinces, the effect of ER on FDI quality is not significant, indicating that the mediation role of FDI quality does not exist in inland China.

Findings from the control variable indicated that when governmental intervention increases, it impedes improvement of FDI quality at the national level, more specifically, in the coastal areas. Infrastructure level plays a positive role on promoting FDI quality as well as GTFP growth at 10% significance level. Massive infrastructural development has the tendency to attract large-scale and highly competitive foreign-funded enterprises to China. Additionally, the results of Sobel, Aroian and Goodman tests on the mediation effect of FDI quality are 1.473, 1.396 and 1.5647, respectively. It suggests that the mediation role of FDI quality is significant in China.

## 5. Conclusions

Based on the provincial dataset during 1998–2018, this study employed the Undesirable-Super-EBM-ML model to measure China’s GTFP growth. The system GMM model was adopted to explore the effects of ER on China’s GTFP from the angle of FDI quantity and FDI quality. 

The empirical results showed that China’s GTFP fluctuated during 1998–2013, then increased dramatically during 2013–2018. GTFP in coastland is larger than that in inland. In addition, ER plays a significant promotion role on GTFP growth in China and coastal areas. The mediation effect of FDIM and FDIQ is verified in the intermediary paths of ER to promote China’s GTFP growth in China and coastal areas.

This paper proposed some policies based on the findings. First, it is imperative to strengthen the environmental regulation intensity. The Chinese government should stress environmental governance and pay attention to the synergy between different types of ER to improve the FDI quality and promote GTFP growth. Furthermore, the technology spillover of FDI quality significantly promotes China’s GTFP growth. It is important for China to stick to the “Opening up” strategy and attract FDI inflow from modern high-tech and service industries. Policymakers should also increase the foreign capital entry threshold and promote FDI quality. Last, the increase in financial development efficiency can promote GTFP growth and FDI quantity. The Chinese government should transcend to promote financial development, especially green finance.

### Future Research

This study contributes to the existing literature by analyzing the mediating role of FDI quantity and FDI quality in the influencing channel of environmental regulation on GTFP growth. Due to data unavailability, the data period was restricted from 1998 to 2018, and the provincial data were used. It might be insightful to check the effect of ER on GTFP at a city level or Micro-enterprise level. Furthermore, future research could explore more intermediary paths of ER on green development, e.g., industrial structure upgrading and technological innovation. Future research could also analyze the mechanisms of different environmental regulation tools on GTFP.

## Figures and Tables

**Table 1:** Input–output variables.

Primary Indices	Secondary-Class Indices	Third-Class Indices
Inputs	Labor	Total number of year-end labors (unit: 10,000 person)
	Capital	Capital stock measured by the perpetual inventory method at the base year 1997 (unit: 100 million yuan)
	Energy	Total energy consumption (unit: 10,000 tons of standard coal)
Desirable output	GDP	GDP is deflated at the 1997 price (unit: 100 million yuan)
Undesirable output	CO_2_ emissions	CO_2_ emissions calculated based on IPCC [40] (unit: 10,000 tons)

**Table 2:** Descriptive statistics.

	Variable	Observations	Mean	Standard Deviation	Min	Max
Input	*Labor* *_it_*	660	462.172	306.788	42.500	1994.137
	*Capital* *_it_*	660	25,976.550	27,277.780	881	157,714.100
	*Energy* *_it_*	660	10,384.300	7829.170	390	40,581
Desirable output	*GDP* *_it_*	660	3114.216	2328.163	202.050	11,601.130
Undesirable output	*CO_2_* *_it_*	660	30,416.410	25,421.640	668.061	152,567.400
Dependent variable	*GTFP* *_it_*	630	0.955	0.062	0.718	1.276
Key Variable	*ER* *_it_*	630	0.180	0.190	0.0001	0.999
Mediation variable	*FDIS* *_it_*	630	0.461	0.574	0.047	5.705
	*FDIQ* *_it_*	630	0.033	0.035	0.001	0.184
Control variable	*Fine* *_it_*	630	0.779	0.147	0.449	1.584
	*Gov* *_it_*	630	0.193	0.093	0.058	0.627
	*Infra* *_it_*	630	0.051	0.024	0.014	0.152

**Table 3:** The values of ML index under metafrontier for 30 provinces in China (1998–2018).

Coastal Area				Inland Area							
	ML	EC	TC		ML	EC	TC		ML	EC	TC
Tianjin	0.981	1.008	0.973	Beijing	1.009	1.043	0.968	Sichuan	0.960	1.003	0.957
Hebei	0.974	1.023	0.955	Shanxi	0.957	1.003	0.954	Guizhou	0.935	1.003	0.932
Liaoning	0.950	1.009	0.946	Inner Mongolia	0.941	0.990	0.954	Yunnan	0.950	0.998	0.952
Shanghai	0.998	1.018	0.980	Jilin	0.933	1.009	0.928	Shaanxi	0.954	0.999	0.955
Jiangsu	0.990	1.007	0.983	Heilongjiang	0.928	1.001	0.928	Gansu	0.856	0.999	0.859
Zhejiang	0.980	1.000	0.981	Anhui	0.944	0.997	0.948	Qinghai	0.966	1.002	0.964
Fujian	0.953	0.982	0.970	Jiangxi	0.936	0.999	0.938	Ningxia	0.925	1.002	0.924
Shandong	0.962	1.004	0.964	Henan	0.955	1.000	0.956	Xinjiang	0.953	1.007	0.946
Guangdong	0.992	1.001	0.991	Hubei	0.967	1.008	0.960				
Guangxi	0.935	0.985	0.950	Hunan	0.960	1.000	0.960				
Hainan	0.949	0.984	0.966	Chongqing	0.960	0.998	0.962				
Mean	0.969	1.002	0.969					Mean	0.947	1.003	0.944

**Table 4:** Multiple collinearity test.

Variable		*GTFP_i(t__−1)_*	*ER_it_*	*FDI_it_*	*Fine_it_*	*Gov_it_*	*Infra_it_*	Mean VIF
*FDIM_it_*	VIF	1.07	1.27	1.11	1.09	1.28	1.07	1.15
	1/VIF	0.939	0.786	0.897	0.914	0.783	0.932	
*FDIQ_it_*	VIF	1.08	1.81	1.74	1.10	1.26	1.05	1.34
	1/VIF	0.923	0.552	0.574	0.909	0.795	0.948	

**Table 5:** Panel unit root test.

		IPS		Breitung		ADF	
		Statistics	*p*-Value	Statistics	*p*-Value	Statistics	*p*-Value
At level	*GTFP_it_*	−8.420 ***	0.000	−7.500 ***	0.000	18.934 ***	0.000
	*ER_it_*	−5.427 ***	0.000	−8.202 ***	0.000	9.032 ***	0.000
	*FDIM_it_*	−2.615 ***	0.005	−0.559	0.288	3.172 ***	0.001
	*FDIQ_it_*	−0.923	0.178	−0.281	0.390	3.699 ***	0.000
	*Fine_it_*	−3.549 ***	0.000	0.428 ***	0.666	11.259 ***	0.000
	*Gov_it_*	3.236	0.999	7.999	1.000	−3.042	0.999
	*Infra_it_*	−0.755	0.225	−2.405 ***	0.008	−1.340	0.910
At first difference	*GTFP_it_*	−14.835 ***	0.000	−12.326 ***	0.000	82.665 ***	0.000
	*ER_it_*	−14.167 ***	0.000	−12.674 ***	0.000	76.493 ***	0.000
	*FDIM_it_*	−7.941 ***	0.000	−5.233 ***	0.000	23.011 ***	0.000
	*FDIQ_it_*	−12.007 ***	0.000	−9.275 ***	0.000	48.763 ***	0.000
	*Fine_it_*	−8.704 ***	0.000	−7.439 ***	0.000	21.735 ***	0.000
	*Gov_it_*	−11.269 ***	0.000	−12.864 ***	0.000	35.745 ***	0.000
	*Infra_it_*	−6.664 ***	0.000	−12.026 ***	0.000	10.707 ***	0.000

Note: *** *p* < 0.01.

**Table 6:** Panel cointegration test.

	Kao Test		Pedroni Test		Westerlund Test	
	Statistic	Value	Statistic	Value	Statistic	Value
*FDIM_it_*	Modified DF	−16.124 ***	Modified PP	4.812 ***	Variance ratio	−1.982 ***
	DF	−16.355 ***	PP	−11.350 ***		
	Augmented DF	−10.828 ***	Augmented DF	−12.792 ***		
	Unadjusted modified DF	−25.545 ***				
	Unadjusted DF	−18.0579 ***				
*FDIQ_it_*	Modified DF	−7.831 ***	Modified PP	4.727 ***	Variance ratio	−1.924 ***
	DF	−12.321 ***	PP	−10.191 ***		
	Augmented DF	−7.248 ***	Augmented DF	−10.788 ***		
	Unadjusted modified DF	−25.553 ***				
	Unadjusted DF	−18.073 ***				

Note: *** *p* < 0.01. DF denotes Dickey–Fuller; PP denotes Phillips–Perron.

**Table 7:** Direct impact of ER on China’s GTFP.

			Heterogeneity Analysis		Robust Analysis			
	Nation		Coastal	Inland	Tobit	Truncated	*ER_i(t−__1)_*	*ER_i(t−__2)_*
*GTFP_i(t__−1)_*	0.365 ***	0.259 ***	0.176 **	0.401 ***	0.396 ***	0.390 ***	0.239 ***	0.279 ***
	(0.053)	(0.084)	(0.073)	(0.090)	(0.038)	(0.037)	(0.0812)	(0.0853)
*ER_it_*	0.063 **	0.068 **	0.038 *	−0.011	0.030 **	0.033 **	0.0739 **	0.0718 **
	(0.030)	(0.030)	(0.022)	(0.037)	(0.013)	(0.013)	(0.0354)	(0.0285)
*Fine_it_*		0.133 *	0.041	−0.007	0.009	0.016	0.229 ***	0.192 ***
		(0.070)	(0.076)	(0.056)	(0.018)	(0.017)	(0.0718)	(0.0615)
*Gov_it_*		0.089	0.115	−0.025	−0.026	−0.023	0.113	0.0901
		(0.085)	(0.089)	(0.111)	(0.027)	(0.027)	(0.1090)	(0.0963)
*Infra_it_*		0.208	0.148	0.068	0.029	0.028	0.310 *	0.255 *
		(0.132)	(0.118)	(0.143)	(0.097)	(0.094)	(0.1610)	(0.1490)
Constant	0.594 ***	0.563 ***	0.731 ***	0.574 ***	0.567 ***	0.566 ***	0.239 ***	0.279 ***
	(0.052)	(0.092)	(0.126)	(0.067)	(0.040)	(0.039)	(0.0812)	(0.0853)
Log likelihood					879.947	898.566		
AR (1)	{0.000}	{0.000}	{0.020}	{0.001}			{0.001}	{0.001}
AR (2)	{0.164}	{0.128}	{0.125}	{0.545}			{0.106}	{0.159}
Hansen	{0.721}	{0.769}	{1.000}	{0.992}			{0.582}	{0.990}
Observations	600	600	220	380	600	600	600	600

Notes: *** *p* < 0.01, ** *p* < 0.05, * *p* < 0.1. The standard errors are in parentheses. *p* values are in braces.

**Table 8:** Indirect impact of ER on China’s GTFP growth: mediation effect of FDI quantity.

	Nation				Coastal		Inland	
	*FDIM_it_*	*GTFP_it_*	*FDIM_it_*	*GTFP_it_*	*FDIM_it_*	*GTFP_it_*	*FDIM_it_*	*GTFP_it_*
*GTFP_i(t−__1)_*		0.278 ***		0.291 ***		0.253 *		0.135
		(0.099)		(0.078)		(0.135)		(0.124)
*FDIM_i(t−__1)_*	0.896 ***		0.926 ***		0.923 ***		1.036 ***	
	(0.054)		(0.073)		(0.065)		(0.074)	
*ER_it_*	0.096	0.026	0.170 ***	0.057 **	0.200 ***	0.091	0.001	−0.055
	(0.066)	(0.017)	(0.053)	(0.024)	(0.071)	(0.062)	(0.075)	(0.057)
*FDIM_it_*		0.029 **		0.033 **		0.043 *		0.2290 *
		(0.013)		(0.013)		(0.023)		(0.134)
*Fine_it_*			0.232 **	0.066	0.433	−0.030	0.043	−0.060
			(0.114)	(0.052)	(0.286)	(0.139)	(0.038)	(0.056)
*Gov_it_*			0.393	0.056	0.650	0.383 *	0.339 **	0.039
			(0.311)	(0.079)	(0.530)	(0.213)	(0.143)	(0.122)
*Infra_it_*			−0.081	−0.006	−0.263	−0.052	−0.1050	−0.184
			(0.273)	(0.105)	(0.532)	(0.085)	(0.203)	(0.120)
Constant	0.020	0.671 ***	−0.259 **	0.589 ***	−0.432 **	0.630 ***	−0.114 **	0.817 ***
	(0.031)	(0.095)	(0.119)	(0.094)	(0.208)	(0.228)	(0.047)	(0.099)
AR (1)	{0.292}	{0.000}	{0.281}	{0.000}	{0.296}	{0.029}	{0.015}	{0.009}
AR (2)	{0.325}	{0.144}	{0.328}	{0.169}	{0.324}	{0.193}	{0.253}	{0.339}
Hansen	{0.852}	{0.762}	{0.911}	{1.000}	{1.000}	{1.000}	{0.998}	{0.985}
Observations	600	600	600	600	220	220	380	380

Notes: *** *p* < 0.01, ** *p* < 0.05, * *p* < 0.1. The standard errors are in parentheses. *p* values are in braces.

**Table 9:** Indirect impact of ER on China’s GTFP growth: mediation effect of FDI quality.

	Nation				Coastal		Inland	
	*FDIQ_it_*	*GTFP_it_*	*FDIQ_it_*	*GTFP_it_*	*FDIQ_it_*	*GTFP_it_*	*FDIQ_it_*	*GTFP_it_*
*GTFP_i(t−__1)_*		0.305 ***		0.280 ***		0.173 **		0.147
		(0.085)		(0.099)		(0.077)		(0.106)
*FDIQ_i(t−__1)_*	0.929 ***		0.885 ***		0.903 ***		0.982 ***	
	(0.02)		(0.043)		(0.063)		(0.064)	
*ER_it_*	0.006 *	−0.007	0.009 **	0.005	0.009 **	0.042 **	0.004	−0.083
	(0.003)	(0.019)	(0.004)	(0.021)	(0.004)	(0.020)	(0.006)	(0.059)
*FDIQ_it_*		0.419 ***		0.405 **		0.299 *		3.691 ***
		(0.160)		(0.206)		(0.164)		(1.386)
*Fine_it_*			−0.002	0.039	0.018	0.078	0.005	0.043
			(0.014)	(0.066)	(0.023)	(0.086)	(0.003)	(0.049)
*Gov_it_*			−0.017 **	−0.002	−0.022 *	0.169 *	0.006	−0.027
			(0.007)	(0.106)	(0.012)	(0.100)	(0.006)	(0.094)
*Infra_it_*			0.018 **	0.205 *	0.040 ***	−0.027	−0.002	0.098
			(0.008)	(0.116)	(0.014)	(0.102)	(0.016)	(0.145)
Constant	0.001 **	0.649 ***	0.006	0.632 ***	−0.009	0.6860 ***	−0.005	0.723 ***
	(0.001)	(0.082)	(0.011)	(0.080)	(0.021)	(0.133)	(0.004)	(0.083)
AR (1)	{0.109}	{0.000}	{0.114}	{0.001}	{0.222}	{0.020}	{0.035}	{0.005}
AR (2)	{0.208}	{0.132}	{0.208}	{0.121}	{0.219}	{0.118}	{0.478}	{0.495}
Hansen	{1.000}	{0.772}	{0.897}	{0.536}	{1.000}	{1.000}	{0.999}	{0.992}
Observations	600	600	600	600	220	220	380	380

Notes: *** *p* < 0.01, ** *p* < 0.05, * *p* < 0.1. The standard errors are in parentheses. *p* values are in braces.

## Data Availability

Data used in this paper are available in China Statistical Yearbook, China Environmental Statistical Yearbook.
